# Two Different Bacterial Community Types Are Linked with the Low-Methane Emission Trait in Sheep

**DOI:** 10.1371/journal.pone.0103171

**Published:** 2014-07-31

**Authors:** Sandra Kittelmann, Cesar S. Pinares-Patiño, Henning Seedorf, Michelle R. Kirk, Siva Ganesh, John C. McEwan, Peter H. Janssen

**Affiliations:** 1 AgResearch Ltd., Grasslands Research Centre, Palmerston North, New Zealand; 2 AgResearch Ltd., Invermay Agricultural Centre, Mosgiel, New Zealand; Wageningen University, Netherlands

## Abstract

The potent greenhouse gas methane (CH_4_) is produced in the rumens of ruminant animals from hydrogen produced during microbial degradation of ingested feed. The natural animal-to-animal variation in the amount of CH_4_ emitted and the heritability of this trait offer a means for reducing CH_4_ emissions by selecting low-CH_4_ emitting animals for breeding. We demonstrate that differences in rumen microbial community structure are linked to high and low CH_4_ emissions in sheep. Bacterial community structures in 236 rumen samples from 118 high- and low-CH_4_ emitting sheep formed gradual transitions between three ruminotypes. Two of these (Q and S) were linked to significantly lower CH_4_ yields (14.4 and 13.6 g CH_4_/kg dry matter intake [DMI], respectively) than the third type (H; 15.9 g CH_4_/kg DMI; *p*<0.001). Low-CH_4_ ruminotype Q was associated with a significantly lower ruminal acetate to propionate ratio (3.7±0.4) than S (4.4±0.7; *p*<0.001) and H (4.3±0.5; *p*<0.001), and harbored high relative abundances of the propionate-producing *Quinella ovalis*. Low-CH_4_ ruminotype S was characterized by lactate- and succinate-producing *Fibrobacter* spp., *Kandleria vitulina*, *Olsenella* spp., *Prevotella bryantii*, and *Sharpea azabuensis*. High-CH_4_ ruminotype H had higher relative abundances of species belonging to *Ruminococcus,* other Ruminococcaceae, Lachnospiraceae, Catabacteriaceae, *Coprococcus*, other Clostridiales, *Prevotella*, other Bacteroidales, and Alphaproteobacteria, many of which are known to form significant amounts of hydrogen. We hypothesize that lower CH_4_ yields are the result of bacterial communities that ferment ingested feed to relatively less hydrogen, which results in less CH_4_ being formed.

## Introduction

The forestomachs of ruminant animals contain a great diversity of prokaryotic and eukaryotic microorganisms that together break down and ferment the feed ingested by the host animal. Volatile fatty acids (VFAs), such as acetate, propionate and butyrate, are formed, together with varying amounts of hydrogen (H_2_). Methanogenic archaea in the rumen use H_2_ to gain energy, producing methane (CH_4_) in the process. CH_4_ is of no nutritional value to the animal, and is eructed and exhaled into the atmosphere, where it acts as a potent greenhouse gas. This CH_4_ also represents a major loss of energy to the animal [1], [2]. To reduce CH_4_ emissions from enteric fermentation, and increase animal productivity, a number of different mitigation strategies have been tested, e.g., feed supplementation with lipids [3], [4], [5], inhibition of enzymes involved in CH_4_ formation [6], [7], depletion of ciliate protozoa [8], or vaccination against methanogens (for a recent review see Wedlock *et al.* [9]). Another potentially very effective way to reduce CH_4_ emissions from ruminant animals is to specifically select naturally low-CH_4_ emitting animals for breeding and to avoid proliferation of high-CH_4_ emitting animals. Measurements of CH_4_ emissions from individual sheep in highly-sensitive open-circuit respiration chambers showed that animals in the same flock, even though feeding on the same diet, varied significantly and consistently in their CH_4_ yields, measured in g CH_4_ per kg of dry matter intake (DMI; [10]). Some individuals have a naturally lower CH_4_ yield (low emitters) than others (high emitters). The genetics of the low CH_4_ trait, including estimates of heritability, repeatability and genetic correlations with productive traits, are starting to be better understood [11]. Of increasing interest are the underlying factors, both genetic and non-genetic, that explain the observed natural differences in CH_4_ yields between individual animals. It is assumed that certain host-related characteristics, such as genotype, physiological state, or development of the animal, influence CH_4_ yields by controlling the presence and/or abundance of certain microbial populations in the rumen. Studies that analyze the microbiota of ruminants that naturally vary in the amount of CH_4_ produced have so far been missing from the literature, and detailed microbial analyses of hundreds of samples have only become possible with the development of next generation sequencing technologies. Understanding the differences in rumen microbial community structure between low- and high-emitting animals will point to those microbial groups that play key roles in the expression of the host trait or that have adapted to it. Isolation and cultivation efforts can then be made to study these particular taxa in greater detail in the future. Knowledge on the physiology of these groups may be useful for targeted modification of rumen microbial communities and promotion of the low-CH_4_ trait, or help understand the circumstances that lead to a low-CH_4_ trait and any production benefits or tradeoffs. Here, we applied high-throughput barcoded 454 Titanium amplicon sequencing of bacterial, archaeal, and eukaryotic marker genes to determine correlations between rumen microbial community structure and CH_4_ yields of 60 high- and 58 low-emitting sheep.

## Results

### CH_4_ yields from sheep are subject to significant natural variation

Individual CH_4_ yields from 340 sheep, in four cohorts, were measured using open-circuit respiration chambers. Two full-day measurements were made on each animal on two consecutive days (round [a]), and then again at least 15 days later (round [b]). The CH_4_ yields (g CH_4_/kg of dry matter intake [DMI]) were expressed as the means of the two days of each round, giving two values for each animal, one for round [a] and one for round [b]. High- (Hi) and low- (Lo) CH_4_ emitting animals were identified from each cohort for rumen microbial community analysis, based on their average CH_4_ yields over the two measuring rounds. This grouping, Hi or Lo, is referred to as CH_4_ group (Table 1). In total, 118 animals were selected for study as Hi (60 animals) or Lo emitters (58 animals), and displayed highly significant differences in CH_4_ yields (*p*<0.001, Student’s t-test). These differences were stable over the two measuring rounds for each animal (*p*<0.001; Table 2). These two sets of animals with distinctly different CH_4_ yields were used to determine if there were parallel differences in rumen microbial community structure based on bacterial, archaeal, and ciliate small subunit rRNA genes and anaerobic fungal internal transcribed spacer 1 (ITS1) genes. Two rumen samples, one from each of the two CH_4_ measurement rounds [a] and [b], were investigated from each of the 118 animals, resulting in microbial community structure data from up to 236 rumen samples per microbial group (Table 2). An overview of microbial community composition and diversity across all analyzed samples is shown in Figure S1 and Figure S2, respectively. Each sample was associated with a CH_4_ yield for the particular sheep and round from which it came.

**Table 1:** Abbreviations used for groups of animals and groups of rumen samples.

Grouping	Basis	Abbreviation			Name of column in File S1
		High-CH_4_	Low-CH_4_		
CH_4_ group	Animals by 4-day average CH_4_ yield	Hi	Lo		CH4Group
CA type	Samples grouped by CA coordinates [Bacteria]	HM	LM1	LM2	CAType
Ruminotype	Designated ruminotype [Bacteria]	H	Q	S	Ruminotype

Groups of animals and rumen samples were classified based on CH_4_ yield only, or based on CH_4_ yield and clustering in correspondence analysis, and corresponding abbreviations of designated ruminotypes.

**Table 2:** Overview of animals screened for CH_4_ yields and rumen samples collected for microbial community structure analysis.

Cohort	1	2	3	4
Measuring rounds	[a] and [b]	[a] and [b]	[a] and [b]	[a] and [b]
No. of animals screened	95	96	101	48
No. of animals analyzed	28	30	30	30
No. of samples analyzed	56	60	60	60
Average CH_4_ yield ± StDev				
Hi emitters	16.9±1.6	17.8±1.2	16.6±1.1	15.6±1.1
Lo emitters	12.0±1.6	14.5±0.9	12.9±1.2	13.5±0.7
*p* [a] Hi *versus* Lo emitters	6.7×10^−10^	1.4×10^−8^	8.4×10^−10^	1.3×10^−5^
*p* [b] Hi *versus* Lo emitters	5.4×10^−8^	3.8×10^−11^	4.8×10^−10^	7.8×10^−10^
*p* [a][b] Hi *versus* Lo emitters	5.5×10^−16^	1.9×10^−17^	5.6×10^−18^	1.7×10^−12^

Average CH_4_ yields and standard deviations (StDev) in Hi and Lo emitters are given in g CH_4_/kg DMI. Probability values were calculated to test for significance between animals with contrasting CH_4_ traits (Student’s t-test).

### High and low CH_4_ sheep harbor similar numbers of methanogenic archaea

**Figure 1:**
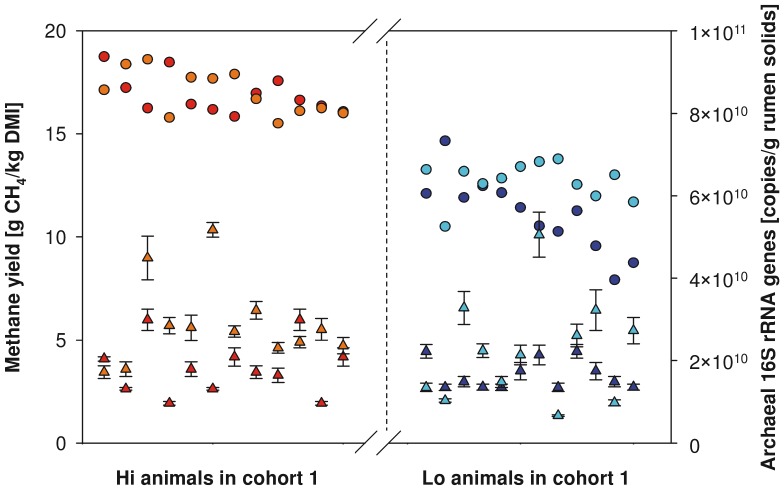
Densities of archaeal 16S rRNA genes in high- and low-CH_4_ emitting sheep. Archaeal 16S rRNA genes (triangles) were quantified in 12 randomly-selected Hi and 12 randomly-selected Lo emitters of cohort 1, and are shown with their corresponding average CH_4_ yields (dots; each the mean of two consecutive days of measurement). Samples are ranked by average CH_4_ yield. Red triangles = number of archaeal 16S rRNA genes per g freeze-dried rumen content in Hi emitters collected in measuring round [a], orange triangles = number of archaeal 16S rRNA genes per g freeze-dried rumen content in Hi emitters collected in measuring round [b], blue triangles = number of archaeal 16S rRNA genes per g freeze-dried rumen content in Lo emitters collected in measuring round [a], light blue triangles = number of archaeal 16S rRNA genes per g freeze-dried rumen content in Lo emitters collected in measuring round [b]. Red dots = average CH_4_ yields of Hi emitters in measuring round [a], orange dots = average CH_4_ yields of Hi emitters in measuring round [b], blue dots = average CH_4_ yields of Lo emitters in measuring round [a], light blue dots = average CH_4_ yields of Lo emitters in measuring round [b]. Error bars represent the standard deviations of four technical replicates of quantitative PCR.

We enumerated archaeal 16S rRNA genes by quantitative PCR to find out if differences in CH_4_ yield correlated with differences in methanogen densities, on the assumption that all archaea present at any significant levels in the rumen are methanogens [12], [13]. However, archaeal 16S rRNA gene abundance was not significantly different in rumen samples from 12 Hi and 12 Lo emitters in both measuring rounds of cohort 1 (*p = *0.91 and *p* = 0.14 for rounds [a] and [b], respectively; Wilcoxon rank-sum test; Figure 1).

### Two different bacterial community types are linked to lower CH_4_ yields in sheep

**Figure 2:**
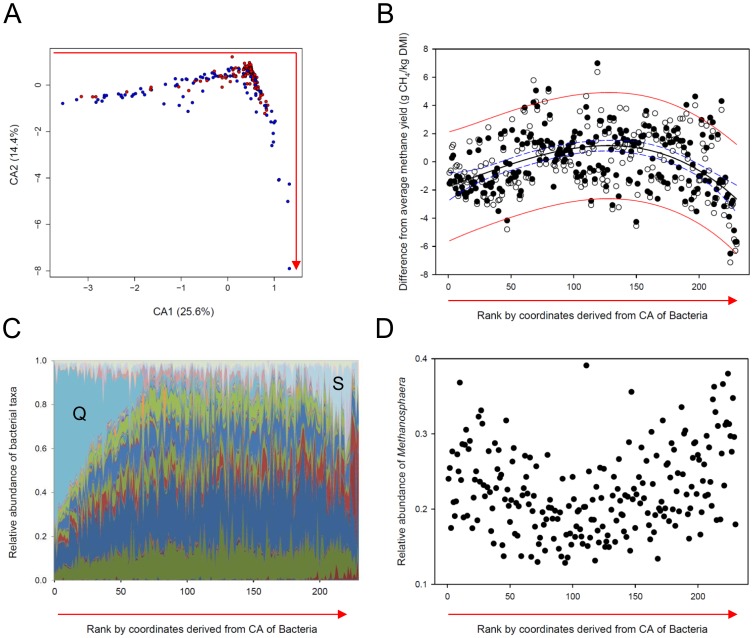
Correlation of rumen microbial community structure with CH_4_ yields of sheep. (A) Correspondence analysis of bacterial communities in 230 rumen samples revealed a relative abundance gradient of taxa across all samples. Samples of animals ranked as Lo emitters were represented more strongly at both tips of the graph, whereas samples of animals ranked as Hi emitters grouped more frequently in the center. (B) Differences of individual CH_4_ yields associated with each sample (n = 230) from the average CH_4_ yield for all samples (○) or for samples within each measuring round (•). A cubic polynomial function was fitted to the within measuring round data (black solid line), and 95% confidence and prediction bands are indicated as dashed blue and solid red lines, respectively. The samples are ordered from left to right corresponding to the order along the red arrow shown in panel (A). (C) Area plot of relative abundances of bacterial taxa in the 230 rumen samples sorted from left to right along the red arrow in panel (A) from top left to bottom right. The relative community composition in each sample is indicated by the colored segments. Q = *Quinella ovalis*, S = *Sharpea azabuensis*. For a detailed color key see Figure S5. (D) Relative abundance of species belonging to the archaeal genus *Methanosphaera* in 226 rumen samples of Hi and Lo emitters plotted from left to right along the red arrow in panel (A).

Differences in bacterial community composition between animals ranked as high-CH_4_ emitters and those ranked as low-CH_4_ emitters was explored using correspondence analysis (CA; Figure 2A). The resultant plot shows a continuous pattern of samples in a right angle arrangement, indicating a gradient of bacterial community structure across all samples. While samples obtained from animals ranked as high-CH_4_ emitters clustered predominantly in and around the center of the graph, samples from animals ranked as low-CH_4_ emitters appeared to predominantly cluster along the horizontal and vertical branches. Canonical discriminant analysis (CDA) corroborated the finding of significant differences between bacterial communities in the rumens of low- and high-CH_4_ emitting animals (*p*<0.001; Figure S3A). Based on results obtained from CA, samples grouping on the horizontal branch (samples 1–67; average CH_4_ yield: 14.4±1.8 g CH_4_/kg DMI) and vertical branch (samples 194–230, 13.6±2.8 g CH_4_/kg DMI) were associated with significantly lower CH_4_ yields than samples clustering in the center (samples 68–193; 15.9±2.0 g CH_4_/kg DMI; Student’s t-test for horizontal branch *versus* center: *p*<0.001; Student’s t-test for vertical branch *versus* center: *p*<0.001; Figure 2B).

We confirmed this finding by performing principal coordinate analysis (PCoA) on the same data, which revealed the same pattern of sample relationships (Figure S4A). As found in the CA, samples towards the extremes of the branches were associated with low CH_4_ yields and samples at the apex of the angle were associated with high CH_4_ yields (Figure S4B). The relative placements of the samples using CA and PCoA were highly correlated (R^2^ = 0.94), showing that both ordination methods gave highly comparable results (Figure S4C). We then used Partitioning Around Medoids to naïvely divide the samples in the PCoA into three clusters based on minimization of dissimilarity of all samples in a cluster to the center of that cluster (Figure S4A). The resulting clusters 2 (14.8±2.6 g CH_4_/kg DMI) and 3 (14.1±1.4 g CH_4_/kg DMI) were characterized by significantly lower average CH_4_ yields than cluster 1 (15.6±2.1 g CH_4_/kg DMI; Student’s t-test cluster 2 *versus* cluster 1: *p* = 0.02; Student’s t-test cluster 3 *versus* cluster 1: *p*<0.001). These analyses all show that at least two different bacterial community types are associated with low-CH_4_ yields and one bacterial community type is associated with high-CH_4_ yields. Clear linear relationships between CH_4_ yield and bacterial community structure were not found (Table S1). This is because key low-CH_4_ taxa can be either abundant or rare in a sample depending on which of the two low-CH_4_ community types was present. Based on the results from the CA, which are supported by the PCoA, we categorized samples 1–67, based on their CA coordinates, as low-CH_4_-associated CA type LM1, samples 68–193 as high-CH_4_-associated CA type HM, and samples 194–230 as low-CH_4_-associated CA type LM2 (Table 1). In addition to lower CH_4_ yields, LM1 samples were also associated with significantly lower acetate to propionate ratios (LM1 average acetate to propionate ratio: 3.7±0.4, HM: 4.3±0.5, LM2∶4.4±0.7; Student’s t-test LM1 *versus* HM: *p*<0.001, Student’s t-test LM2 *versus* HM: *p* = 0.83).

Notably, the three different CA community types LM1, LM2, and HM were each characterized by a higher relative abundance of a number of key bacterial taxa (Figure 2C, Figure S5). ANOVA based on the three CA types revealed the taxon composition of samples belonging to each category (Table 3), and three “ruminotypes” were defined (Table 1; for ANOVA results based on PAM clustering see Table S2). Of the taxa that represented on average ≥1% of the bacterial community in at least one of the three ruminotypes, larger abundances of species belonging to the Ruminococcaceae, Clostridiales, Lachnospiraceae, *Prevotella*, Bacteroidales, Alphaproteobacteria, Catabacteriaceae, *Coprococcus*, and *Ruminococcus* (in order of significance) were characteristic of ruminotype H (H = HM-associated). Species of the genus *Quinella* were most abundant in LM1-associated ruminotype Q (Q = *Quinella ovalis*). Sequences belonging to the genus *Quinella* were initially assigned to the genus *Selenomonas* with unknown species affiliation due to the lack of a reference sequence for the genus *Quinella* in the greengenes database that we used. A BLAST search against the GenBank database and comparison of the *Selenomonas*-assigned sequences to the 16S rRNA gene sequence of *Quinella ovalis* (Quin’s oval) in ARB revealed their true affiliation with the genus *Quinella*. Quite different taxa (*Sharpea azabuensis*, *Olsenella*, *Fibrobacter succinogenes*, *Prevotella bryantii*, *Kandleria vitulina*, and *Fibrobacter intestinalis*) contributed predominantly to LM2-associated ruminotype S (S = *Sharpea azabuensis*).

**Table 3:** Analysis of variance between samples grouping into the three different bacterial community types.

Taxon	*p*-value	average relative abundance [%]		
		LM1 [Q]	HM [H]	LM2 [S]
*Quinella*	5.1×10^−54^	32.5	1.6	0.4
*Sharpea azabuensis*	1.7×10^−31^	1.0	1.7	11.9
*Olsenella*	1.3×10^−18^	0.1	0.1	1.8
Ruminococcaceae	2.2×10^−17^	4.3	6.9	3.4
*Fibrobacter succinogenes*	1.5×10^−14^	3.8	4.5	9.1
Clostridiales	3.3×10^−12^	4.8	6.5	3.3
*Prevotella bryantii*	1.6×10^−11^	0.5	0.5	5.7
Lachnospiraceae	2.3×10^−11^	7.0	10.7	8.6
*Prevotella*	9.4×10^−11^	17.6	27.1	22.9
Bacteroidales	3.4×10^−9^	9.2	14.0	11.1
Alphaproteobacteria	1.2×10^−8^	0.9	1.8	0.4
Catabacteriaceae	2.4×10^−7^	1.0	1.6	0.4
*Kandleria vitulina*	7.9×10^−5^	0.4	0.4	2.6
*Coprococcus*	4.7×10^−3^	0.8	1.2	0.9
*Fibrobacter intestinalis*	7.4×10^−3^	0.1	0.1	1.2
*Ruminococcus*	3.2×10^−2^	1.1	1.4	1.0

Bonferroni-corrected *p*-values for significance (at 0.05 criterion) of bacterial taxa were obtained by performing ANOVA on the samples grouping into the three different bacterial community types: LM1 [ruminotype Q], HM [ruminotype H], and LM2 [ruminotype S]. Mean relative abundances of significant bacterial taxa in the three different bacterial community types are presented. Only significant taxa that contributed in average ≥1% to the total bacterial community in at least one community type are shown.

### Relative abundance of *Methanosphaera* spp. is increased in LM samples

Correspondence analysis did not reveal any specific clustering pattern based on archaeal community structure (Figure S6A); however, CDA suggested significant differences between archaeal communities in animals ranked as low and high CH_4_ emitters (*p*<0.001; Figure S3B). To obtain a better understanding of which taxa, if any, were significantly correlated with CH_4_ yield, relative abundances of archaeal taxa were compared with CH_4_ yield in the individual measuring rounds using Spearman’s Rank correlations. This analysis indicated that the relative abundance of *Methanosphaera* spp. was negatively and significantly correlated with CH_4_ yield in several - but not in all - of the eight measuring rounds (Table S1). This finding was confirmed by the results obtained from plotting *Methanosphaera* relative abundance against the ranking of each sample derived from CA on the bacterial data set (Figure 2D). Samples categorized as LM1 and LM2 harbored significantly higher relative abundances of *Methanosphaera* than samples belonging to the HM type (LM1∶0.23±0.05 [average ± standard deviation], LM2∶0.27±0.06, HM: 0.20±0.05; Student’s t-test LM1 *versus* HM: *p*<0.001, Student’s t-test LM2 *versus* HM: *p*<0.001).

### Eukaryotic community structure appears to be independent of CH_4_ yield

The composition of eukaryotic communities in the rumen samples was explored using CA and CDA, but neither ciliate nor anaerobic fungal communities revealed any obvious clustering by CH_4_ group of the animals that could have been attributed to differential CH_4_ yields (Figure S3C, Figure S3D, Figure S6B, Figure S6C). Spearman’s Rank correlations suggested that few eukaryotic taxa were correlated with CH_4_ yield in the different measuring rounds (Table S1). Overall, however, no consistent trends were observed, and for the majority of taxa there was no correlation at all.

## Discussion

This work demonstrates that the natural variation in the CH_4_ emission trait is reflected in the composition of the microbial community in the rumen of the host animal. We analyzed bacterial, archaeal, and eukaryotic communities in rumen samples obtained from sheep identified as naturally high or low emitting animals using the most accurate method of CH_4_ yield measurement to date (respiration chambers). We reasoned that, if there were differences in the microbial communities of sheep emitting different amounts of CH_4_, these differences should be most apparent in comparisons of the highest and lowest emitting animals, and that they should be consistently found in different groups of animals. Our data on the rumen microbial communities from the 60 highest and 58 lowest CH_4_ emitters from four different cohorts of sheep suggest that differences in bacterial and archaeal community structures are associated with a naturally lower CH_4_ yield and are consistent with consequent differences in rumen fermentation.

### Linking bacterial community structure to CH_4_ yields

Profiling of 16S rRNA genes revealed gradients in bacterial community structure across all analyzed rumen samples, and suggested the existence of at least three idealized bacterial community types, referred to as ruminotypes Q, S, and H. Ruminotype H was linked with samples associated with higher than average CH_4_ yields (HM samples) while ruminotypes Q and S were linked to lower than average CH_4_ yields (LM1 and LM2 samples, respectively). Such intestinal bacterial community types that appear to be specifically associated with a certain host trait have previously been referred to as “enterotypes” in humans [14], [15], chimpanzees [16], and mice [17]. However, we observed that the rumen microbiota across samples categorized as LM1, LM2 and HM displayed smooth abundance gradients of key genera without discrete clustering of samples. For convenience, we have adopted the term “ruminotype” for generalized community types, recognizing that there is so far no clear consensus of the enterotype concept [18].

VFA ratios in the rumen samples suggested that proportionally more propionate was present in LM1 samples. This observation is consistent with the finding that LM1-associated ruminotype Q has a higher relative abundance of organisms closely related to *Quinella ovalis* (Quin’s oval; [19], [20]). Quin’s oval appears to ferment sugars to equimolar acetate and propionate [21], which is associated with lower H_2_ formation than other fermentation pathways [22]. LM2-associated ruminotype S harbored significant proportions of species characterized as lactate producers such as *Sharpea azabuensis* [23], *Kandleria vitulina* [24], [25], and *Olsenella* spp. (*O. uli*; [26]) and succinate producers such as *Fibrobacter succinogenes*, *F. intestinalis* [27] and *Prevotella bryantii* [28]. Formation of these products is generally associated with no or low H_2_ production. In contrast, HM-associated ruminotype H was characterized by higher abundances of bacteria characterized as H_2_ producers, notably members of *Ruminococcus* and other Ruminococcaceae, *Coprococcus*, Lachnospiraceae, and other Clostridiales [29], [30].

### Linking archaeal community structure to CH_4_ yields

Methanogen densities were not significantly different in rumen samples from Hi and Lo emitters of cohort 1 across both CH_4_ measuring rounds. These results corroborate previous studies that also showed that densities of methanogens were not significantly different between two groups of feedlot bulls [31] and two groups of lambs [32] that produced significantly different amounts of CH_4_. In contrast to those studies, where differences in CH_4_ emission resulted from the different diets being administered, differences in CH_4_ yields in our study were due to natural animal-to-animal variation. It has been reported that cattle with naturally high and low feed conversion efficiency vary considerably in the amounts of CH_4_ produced [8], [33]. But, similar to the sheep in our study, the densities of methanogens in the rumen fluid did not vary in animals with different feed conversion efficiencies [34]. Hence, natural differences in CH_4_ yields between individual sheep were not due to naturally differing densities of methanogenic archaea in the rumen, i.e., greater densities of methanogens in high CH_4_ animals and lower densities in low CH_4_ animals.

Archaeal community structure, however, was significantly different in rumen samples associated with different CH_4_ yields. Members of the genus *Methanosphaera* were significantly more abundant in LM1 and LM2 samples as opposed to HM samples.

Recent measurements suggest that the rumens of low CH_4_-emitting sheep are significantly smaller than those of high CH_4_-emitting sheep [35]. Both LM-associated ruminotypes, Q and S, were associated with bacteria that would ferment the ingested feed to less H_2_ than the HM-associated ruminotype H. Fermentation of feed to less H_2_ in low CH_4_ animals would support a smaller population of hydrogenotrophic methanogens (like *Methanobrevibacter* spp.), although in a smaller rumen. This could explain why the density of methanogens was not different. In addition to hydrogenotrophic methanogens, other methanogens like *Methanosphaera* spp. use methyl-groups derived from methoxyl-substituents of plant material as a major CH_4_ precursor [36], [37]. The amount of methyl-group derived CH_4_ is probably limited by the availability of the methyl-donors, which is constant in the feed. This methanogenesis from methyl-groups would therefore support similar numbers of *Methanosphaera* spp. in animals of both CH_4_ traits, although they could be expected to be denser in animals with smaller rumens. This would account for the increased relative abundance of *Methanosphaera* spp. in samples related to LM yields in our study.

### No apparent eukaryotic community structure link to CH_4_ yield

Reports on the eukaryotic community composition in the rumen and its correlation with CH_4_ are scarce, and only very recently has a methodology been developed to analyze not only bacterial and archaeal but also ciliate and anaerobic fungal communities in the rumen using next generation sequencing [38]. Although it has been suggested that certain genera of ciliate protozoa may be linked to higher or lower CH_4_ emissions [39], [40], we did not find significant and consistent indications that ciliate community composition contributed to the natural differences in the animals analyzed in our study. Similarly, anaerobic fungal community structure did not appear to be linked to CH_4_ trait. These findings do not rule out potential differences in ciliate and anaerobic fungal communities on a lower taxonomic, the (meta)genomic or transcriptional level.

### Possible synthesis of findings

Our data show that there is a significant correlation between microbial community structure and natural variations in CH_4_ emissions from sheep fed the same diet. The full set of factors that caused the observed differences in rumen microbial community structure in animals with high- and low-CH_4_ yields remains to be elucidated. Genetic, epigenetic or environmental factors are likely to drive the differences in the rumen microbial communities in naturally high- and low-CH_4_ emitting animals. We postulate that the differences in CH_4_ emissions are a result of such factors selecting for different microbial communities, which in turn form different amounts of H_2_ and so different amounts of CH_4_ per unit of feed. High-CH_4_ emitting sheep have larger rumens than low-CH_4_ emitting sheep [35], and correspondingly lower passage rates [35], [41]. Lower passage rates are associated with lower dissolved H_2_ concentrations which select for H_2_ producing bacteria and hence more CH_4_ formation [22]. Genes of the CH_4_ forming pathways of methanogens were found to be more highly expressed relative to gene copy in high-CH_4_ emitting sheep, which was interpreted as a response to lower H_2_ concentrations in high-CH_4_ emitting sheep [42].

Follow-on studies looking at correlations between microbial community structure and animal genetics (e.g., single nucleotide polymorphisms on the host genome) or physiological and anatomical data (e.g., rumen morphology, pH, passage rate, etc.) can begin to determine which factors drive the microbial community structure and ultimately result in different CH_4_ yields. It is possible that the two different LM-associated ruminotypes are controlled by different factors, and it will be of interest to breeders to evaluate whether one of the two bacterial community types, or even the HM-associated ruminotype, has better production characteristics. If microbial communities are controlled by host genetic factors, then selective breeding can steer ruminant populations towards decreased enteric CH_4_ formation. If the pivotal factors originate in the animal’s environment (e.g., behavior, handling, etc.), then animal management may be adopted as a means to maximize the low-CH_4_ trait.

## Materials and Methods

### Measurement of CH_4_ yields from sheep

The use of animals, including welfare, feeding, experimental procedures, and the collection of rumen samples used for this study, was approved by the AgResearch Grasslands Animal Ethics Committee (Application number 11975), and complied with the institutional Codes of Ethical Conduct for the Use of Animals in Research, Testing and Teaching, as prescribed in the New Zealand Animal Welfare Act of 1999 and its amendments. CH_4_ measurements were conducted on a total of 340 New Zealand sheep separated into four cohorts (or lots; [11]). Two independent measuring rounds, each over two days, were made for each individual, producing a total of four days of CH_4_ yield data (g CH_4_/kg DMI). The larger experiment of which these animals were a part is described in detail by Pinares-Patiño *et al.* [11]. For more information also see Text S1.

### Rumen sampling and sample processing

A rumen sample was collected via stomach tubing and immediately stored at −20°C at the end of each of the two CH_4_ measurement rounds (17–18 h after the last feeding). Animals within each separate cohort were ranked on the basis of their 4-day average CH_4_ yield, and rumen samples of in average high- (Hi) and low-CH_4_ emitting animals (Lo) were selected for analysis of rumen microbial community structure (Table 1). In cohort 1, 15 of the 17 highest CH_4_ emitting animals and 13 of the 15 lowest CH_4_ emitting animals were used. In cohort 2, the 15 highest and 15 of the 16 lowest CH_4_ emitting animals were used. Rumen samples from the 15 highest emitting animals and the 15 lowest emitting animals in cohorts 3 and 4 were used. This gave a total of 236 rumen samples from 118 animals (Table 2; for metadata of individual samples refer to File S1). All rumen samples were freeze-dried, homogenized, and stored at −20°C. VFA concentrations were determined from subsamples of rumen contents by gas-liquid chromatography ([43]; Text S1).

### Extraction and amplification of nucleic acids

Nucleic acids were extracted from freeze-dried rumen contents using a combined bead-beating, phenol-chloroform and column purification protocol using the QIAquick 96 PCR purification kit (Qiagen, Hilden, Germany; Text S1; [44]). The abundances of archaeal 16S rRNA genes were quantified in randomly selected rumen samples of 12 Hi emitters and 12 Lo emitters of cohort 1 as described previously (Text S1; [45]).

### Microbial community analyses

PCR amplification of bacterial and archaeal 16S rRNA genes, ciliate 18S rRNA genes, and the anaerobic fungal internal transcribed spacer 1 (ITS1), was performed using methods modified from Kittelmann *et al.* (described in detail in Text S1; [38]). Pyrosequencing was performed on a 454 Life Sciences Genome Sequencer FLX machine (Center for Genome Sciences and Systems Biology, Washington University of St. Louis, USA).

Sequence data were processed and analyzed following the procedure described by Caporaso *et al.* [46]. Sequence data were phylogenetically assigned using BLAST against reference databases (for more detailed information see Text S1; databases are available from the authors upon request). These sequence data have been submitted to the EMBL database under the study accession numbers ERP003779 (Bacteria), ERP003773 (Archaea), ERP003772 (ciliate protozoa), and ERP003764 (anaerobic fungi). All analyses of high-throughput pyrosequencing data were carried out using the QIIME pipeline [46]. Resulting text files were imported into Excel (Microsoft Corp., Redmond, WA, USA) and R (www.r-project.org) for further statistical evaluation. Detailed descriptions of the statistical analyses performed to evaluate potential differences of microbial community structure in animals with contrasting CH_4_ traits are given in Text S1.

## Supporting Information

Figure S1 **Microbial community composition in sheep rumen samples based on the analysis of marker genes.** (A) Bacteria, 16S rRNA genes, (B) archaea, 16S rRNA genes, (C) ciliate protozoa, 18S rRNA genes, and (D) anaerobic fungi, ITS1. Only taxa contributing on average ≥1% to the representative community are shown, and all taxonomic groups contributing less than 1% are summarized as “other” in all four charts.
(DOCX)

Figure S2 **Average diversity of microbial communities in sheep rumen samples.** Diversity of (A) bacterial, (B) archaeal, (C) ciliate, and (D) anaerobic fungal communities in rumen samples obtained from eight measuring rounds was evaluated using Simpson’s index of diversity (1−*λ*). Simpson’s index of diversity (y-axis of each plot) ranges from 0 to 1, with 1 indicating maximum diversity. Whiskers represent the maximum and minimum values excluding outliers (which are indicated as circles).
(DOCX)

Figure S3 **Canonical discriminant analysis score plot based on microbial community structure data.** Canonical discriminant analysis of (A) bacterial communities in 236, (B) archaeal communities in 226, (C) ciliate communities in 235, and (D) anaerobic fungal communities in 232 rumen samples of Hi (red) and Lo (blue) sheep. Since grouping into two groups (Hi and Lo) results in reduction of data to 1 dimension, data points were plotted against a random jitter (y-axis). All potential variation between CH_4_ groups is explained by the first canonical discriminant function (CDF; x-axis).
(DOCX)

Figure S4 **Correlation of bacterial community structure with CH_4_ yields associated with 230 sheep rumen samples.** (A) Principal coordinate analysis of bacterial communities in rumen samples using the Bray-Curtis dissimilarity metric confirms the relative abundance gradient observed using CA (Figure 2). Partitioning Around Medoids (PAM) was performed to obtain three bacterial community groupings. On the plot, the designations 1 ( =  cluster 1), 2 ( =  cluster 2), and 3 ( =  cluster 3) represent the clusters into which each of the samples grouped using PAM. (B) Differences of individual CH_4_ yields per sample ordered from left to right along the red arrow in panel (A) from the average CH_4_ yield across all samples (○) or across samples within each measuring round (•). A cubic polynomial function was fitted to the within-measuring round data (black solid line) and 95% confidence and prediction bands are indicated as dashed blue and solid red lines, respectively. (C) Correlation of sample ranks derived from PCoA and CA. The samples are ordered from left to right in the same order as along the red arrow in panel (A). A linear function was fitted (solid black line) and gave R^2^ = 0.94.
(DOCX)

Figure S5 **Area plot of relative abundances of bacterial taxa in the 230 rumen samples.** Samples are sorted from left to right along the arrow in Figure 2 panel (A) from top left to bottom right. The relative community composition in each sample is indicated by the colored segments. Q = *Quinella ovalis*, S = *Sharpea azabuensis*. The colors are shown, in the same order, in the key. Ord. = order, fam. = family, gen. = genus, sp. = species, affil. = affiliation.
(DOCX)

Figure S6 **Correspondence analysis based on microbial community structure.** Correspondence analysis of (A) archaeal communities in 226 rumen samples, (B) ciliate communities in 235 rumen samples, and (C) anaerobic fungal communities in 232 rumen samples distinguished based on the CH_4_ group of the animal that the sample originated from (average CH_4_ yield across both measuring rounds). Hi = red, Lo = blue.
(DOCX)

Table S1 **Correlations between microbial taxa and CH_4_ ranks of sheep.** Spearman’s rank correlation factors (ρ) and corresponding *p*-values are shown for those microbial groups that represented at least 1% of the bacterial community in at least one rumen sample in the analyzed measuring round *and* that were found significantly related to either high-CH_4_ or low-CH_4_ in at least one measuring round (rank 1 = sample with highest CH_4_ yield in each measuring round). Taxa that showed a positive correlation in one measuring round and a negative correlation in another were regarded to have no clear correlation with CH_4_ (None). The table is sorted by the number of measuring rounds in which the taxa were found to be significantly correlated to CH_4_ rank (last column (Number of rounds); shaded from dark orange (large number of measuring rounds that showed significant correlation) to light yellow (small number)) and by the type of correlation (second to last column (High/Low; for correlation to high-CH_4_ or low-CH_4_ yields). Here, the taxon *Eubacterium* refers to members of the genus *Eubacterium* which belong to the family *Ruminococcaceae* (according to the greengenes taxonomy).
(DOCX)

Table S2 **Analysis of variance between samples grouping into the three different bacterial community types based on PAM clustering.** Bonferroni-corrected *p*-values obtained by performing ANOVA for significance (at 0.05 criterion) of bacterial taxa between the community clusters defined by PAM and mean relative abundances of significant bacterial taxa in the samples grouping into clusters 1 [ruminotype H], 2 [ruminotype S], and 3 [ruminotype Q]. Only significant taxa that contributed in average ≥1% to the total bacterial community in at least one community type are reported.
(DOCX)

Text S1 **Detailed Materials and Methods.**
(DOCX)

Text S2 **Microbial community composition across all samples.**
(DOCX)

Text S3 **Microbial community diversity across all samples.**
(DOCX)

File S1 (XLS)
