# Supplementary material for: Two Different Bacterial Community Types Are Linked with the Low-Methane Emission Trait in Sheep
Source: PLoS One. 2014 Jul 31;9(7):e103171. doi: 10.1371/journal.pone.0103171 (PMC4117531; doi:10.1371/journal.pone.0103171)
Supplement: Figure S1 — Microbial community composition in sheep rumen samples based on the analysis of marker genes. (A) Bacteria, 16S rRNA genes, (B) archaea, 16S rRNA genes, (C) ciliate protozoa, 18S rRNA genes, and (D) anaerobic fungi, ITS1. Only taxa contributing on average ≥1% to the representative community are shown, and all taxonomic groups contributing less than 1% are summarized as “other” in all four charts. (DOCX) [file pone.0103171.s001.docx]

**Figure S1.** **Microbial community composition in sheep rumen samples based on the analysis of marker genes.** (A) Bacteria, 16S rRNA genes, (B) archaea, 16S rRNA genes, (C) ciliate protozoa, 18S rRNA genes, and (D) anaerobic fungi, ITS1. Only taxa contributing on average ≥1% to the representative community are shown, and all taxonomic groups contributing less than 1% are summarized as “other” in all four charts.
