# Supplementary material for: Two Different Bacterial Community Types Are Linked with the Low-Methane Emission Trait in Sheep
Source: PLoS One. 2014 Jul 31;9(7):e103171. doi: 10.1371/journal.pone.0103171 (PMC4117531; doi:10.1371/journal.pone.0103171)
Supplement: Figure S2 — Average diversity of microbial communities in sheep rumen samples. Diversity of (A) bacterial, (B) archaeal, (C) ciliate, and (D) anaerobic fungal communities in rumen samples obtained from eight measuring rounds was evaluated using Simpson’s index of diversity (1−λ). Simpson’s index of diversity (y-axis of each plot) ranges from 0 to 1, with 1 indicating maximum diversity. Whiskers represent the maximum and minimum values excluding outliers (which are indicated as circles). (DOCX) [file pone.0103171.s002.docx]

**Figure S2.** **Average diversity of microbial communities in sheep rumen samples.** Diversity of (A) bacterial, (B) archaeal, (C) ciliate, and (D) anaerobic fungal communities in rumen samples obtained from eight measuring rounds was evaluated using Simpson’s index of diversity (1-*λ*). Simpson’s index of diversity (y-axis of each plot) ranges from 0 to 1, with 1 indicating maximum diversity. Whiskers represent the maximum and minimum values excluding outliers (which are indicated as circles).
