# Supplementary material for: Two Different Bacterial Community Types Are Linked with the Low-Methane Emission Trait in Sheep
Source: PLoS One. 2014 Jul 31;9(7):e103171. doi: 10.1371/journal.pone.0103171 (PMC4117531; doi:10.1371/journal.pone.0103171)
Supplement: Figure S3 — Canonical discriminant analysis score plot based on microbial community structure data. Canonical discriminant analysis of (A) bacterial communities in 236, (B) archaeal communities in 226, (C) ciliate communities in 235, and (D) anaerobic fungal communities in 232 rumen samples of Hi (red) and Lo (blue) sheep. Since grouping into two groups (Hi and Lo) results in reduction of data to 1 dimension, data points were plotted against a random jitter (y-axis). All potential variation between CH4 groups is explained by the first canonical discriminant function (CDF; x-axis). (DOCX) [file pone.0103171.s003.docx]

**Figure S3. Canonical discriminant analysis score plot based on microbial community structure data.** Canonical discriminant analysis of (A) bacterial communities in 236, (B) archaeal communities in 226, (C) ciliate communities in 235, and (D) anaerobic fungal communities in 232 rumen samples of Hi (red) and Lo (blue) sheep. Since grouping into two groups (Hi and Lo) results in reduction of data to 1 dimension, data points were plotted against a random jitter (y-axis). All potential variation between CH_4_ groups is explained by the first canonical discriminant function (CDF; x-axis).
