# Supplementary material for: Two Different Bacterial Community Types Are Linked with the Low-Methane Emission Trait in Sheep
Source: PLoS One. 2014 Jul 31;9(7):e103171. doi: 10.1371/journal.pone.0103171 (PMC4117531; doi:10.1371/journal.pone.0103171)
Supplement: Figure S4 — Correlation of bacterial community structure with CH4 yields associated with 230 sheep rumen samples. (A) Principal coordinate analysis of bacterial communities in rumen samples using the Bray-Curtis dissimilarity metric confirms the relative abundance gradient observed using CA (Figure 2). Partitioning Around Medoids (PAM) was performed to obtain three bacterial community groupings. On the plot, the designations 1 ( = cluster 1), 2 ( = cluster 2), and 3 ( = cluster 3) represent the clusters into which each of the samples grouped using PAM. (B) Differences of individual CH4 yields per sample ordered from left to right along the red arrow in panel (A) from the average CH4 yield across all samples (○) or across samples within each measuring round (•). A cubic polynomial function was fitted to the within-measuring round data (black solid line) and 95% confidence and prediction bands are indicated as dashed blue and solid red lines, respectively. (C) Correlation of sample ranks derived from PCoA and CA. The samples are ordered from left to right in the same order as along the red arrow in panel (A). A linear function was fitted (solid black line) and gave R2 = 0.94. (DOCX) [file pone.0103171.s004.docx]

**Figure S4. Correlation of bacterial community structure with CH_4_ yields associated with 230 sheep rumen samples.** (A) Principal coordinate analysis of bacterial communities in rumen samples using the Bray-Curtis dissimilarity metric confirms the relative abundance gradient observed using CA (Figure 2). Partitioning Around Medoids (PAM) was performed to obtain three bacterial community groupings. On the plot, the designations 1 (= cluster 1), 2 (= cluster 2), and 3 (= cluster 3) represent the clusters into which each of the samples grouped using PAM. (B) Differences of individual CH_4_ yields per sample ordered from left to right along the red arrow in panel (A) from the average CH_4_ yield across all samples (○) or across samples within each measuring round (●). A cubic polynomial function was fitted to the within-measuring round data (black solid line) and 95% confidence and prediction bands are indicated as dashed blue and solid red lines, respectively. (C) Correlation of sample ranks derived from PCoA and CA. The samples are ordered from left to right in the same order as along the red arrow in panel (A). A linear function was fitted (solid black line) and gave R^2^ = 0.94.
