# Supplementary material for: Two Different Bacterial Community Types Are Linked with the Low-Methane Emission Trait in Sheep
Source: PLoS One. 2014 Jul 31;9(7):e103171. doi: 10.1371/journal.pone.0103171 (PMC4117531; doi:10.1371/journal.pone.0103171)
Supplement: Figure S5 — Area plot of relative abundances of bacterial taxa in the 230 rumen samples. Samples are sorted from left to right along the arrow in Figure 2 panel (A) from top left to bottom right. The relative community composition in each sample is indicated by the colored segments. Q = Quinella ovalis, S = Sharpea azabuensis. The colors are shown, in the same order, in the key. Ord. = order, fam. = family, gen. = genus, sp. = species, affil. = affiliation. (DOCX) [file pone.0103171.s005.docx]

**Figure S5. Area plot of relative abundances of bacterial taxa in the 230 rumen samples.** Samples are sorted from left to right along the arrow in Figure 2 panel (A) from top left to bottom right. The relative community composition in each sample is indicated by the colored segments. Q = *Quinella ovalis*, S = *Sharpea azabuensis*. The colors are shown, in the same order, in the key. Ord. = order, fam. = family, gen. = genus, sp. = species, affil. = affiliation.
