# Supplementary material for: Two Different Bacterial Community Types Are Linked with the Low-Methane Emission Trait in Sheep
Source: PLoS One. 2014 Jul 31;9(7):e103171. doi: 10.1371/journal.pone.0103171 (PMC4117531; doi:10.1371/journal.pone.0103171)
Supplement: Figure S6 — Correspondence analysis based on microbial community structure. Correspondence analysis of (A) archaeal communities in 226 rumen samples, (B) ciliate communities in 235 rumen samples, and (C) anaerobic fungal communities in 232 rumen samples distinguished based on the CH4 group of the animal that the sample originated from (average CH4 yield across both measuring rounds). Hi = red, Lo = blue. (DOCX) [file pone.0103171.s006.docx]

**Figure S6. Correspondence analysis based on microbial community structure.** Correspondence analysis of (A) archaeal communities in 226 rumen samples, (B) ciliate communities in 235 rumen samples, and (C) anaerobic fungal communities in 232 rumen samples distinguished based on the CH_4_ group of the animal that the sample originated from (average CH_4_ yield across both measuring rounds). Hi = red, Lo = blue.
