# Supplementary material for: Two Different Bacterial Community Types Are Linked with the Low-Methane Emission Trait in Sheep
Source: PLoS One. 2014 Jul 31;9(7):e103171. doi: 10.1371/journal.pone.0103171 (PMC4117531; doi:10.1371/journal.pone.0103171)
Supplement: Table S1 — Correlations between microbial taxa and CH4 ranks of sheep. Spearman’s rank correlation factors (ρ) and corresponding p-values are shown for those microbial groups that represented at least 1% of the bacterial community in at least one rumen sample in the analyzed measuring round and that were found significantly related to either high-CH4 or low-CH4 in at least one measuring round (rank 1 = sample with highest CH4 yield in each measuring round). Taxa that showed a positive correlation in one measuring round and a negative correlation in another were regarded to have no clear correlation with CH4 (None). The table is sorted by the number of measuring rounds in which the taxa were found to be significantly correlated to CH4 rank (last column (Number of rounds); shaded from dark orange (large number of measuring rounds that showed significant correlation) to light yellow (small number)) and by the type of correlation (second to last column (High/Low; for correlation to high-CH4 or low-CH4 yields). Here, the taxon Eubacterium refers to members of the genus Eubacterium which belong to the family Ruminococcaceae (according to the greengenes taxonomy). (DOCX) [file pone.0103171.s007.docx]

**Table S1. Correlations between microbial taxa and CH_4_ ranks of sheep.** Spearman’s rank correlation factors (ρ) and corresponding *p*-values are shown for those microbial groups that represented at least 1% of the bacterial community in at least one rumen sample in the analyzed measuring round *and* that were found significantly related to either high-CH_4_ or low-CH_4_ in at least one measuring round (rank 1=sample with highest CH_4_ yield in each measuring round). Taxa that showed a positive correlation in one measuring round and a negative correlation in another were regarded to have no clear correlation with CH_4_ (None). The table is sorted by the number of measuring rounds in which the taxa were found to be significantly correlated to CH_4_ rank (last column (Number of rounds); shaded from dark orange (large number of measuring rounds that showed significant correlation) to light yellow (small number)) and by the type of correlation (second to last column (High/Low; for correlation to high-CH_4_ or low-CH_4_ yields). Here, the taxon *Eubacterium* refers to members of the genus *Eubacterium* which belong to the family *Ruminococcaceae* (according to the greengenes taxonomy).

| Cohort | 1 | | 2 | | 3 | | 4 | | High/Low | Number of rounds |
| --- | --- | --- | --- | --- | --- | --- | --- | --- | --- | --- |
| Measuring round | (a) | (b) | (a) | (b) | (a) | (b) | (a) | (b) |  |  |
|  | ρ  (*p*-value) | ρ  (*p*-value) | ρ  (*p*-value) | ρ  (*p*-value) | ρ  (*p*-value) | ρ  (*p*-value) | ρ  (*p*-value) | ρ  (*p*-value) |  |  |
| Bacterial taxon |  |  |  |  |  |  |  |  |  |  |
| *Ruminococcaceae* | −0.54 (2.73×10^−3^) |  | −0.56  (1.31×10^−3^) | −0.61  (4.44×10^−4^) | −0.43  (1.74×10^−2^) | −0.86  (2.79×10^−9^) | −0.64  (2.71×10^−4^) | −0.44  (1.93×10^−2^) | High | 7 |
| *Catabacteriaceae* | −0.49  (8.29×10^−3^) |  | −0.54  (2.01×10^−3^) | −0.59  (7.72×10^−4^) | −0.56  (1.28×10^−3^) | −0.60  (6.35×10^−4^) | −0.42  (2.77×10^−2^) |  | High | 6 |
| *Clostridiales* | −0.38  (4.75×10^−2^) |  | −0.37  (4.28×10^−2^) | −0.54  (2.64×10^−3^) |  | −0.64  (1.69×10^−4^) | −0.41  (3.15×10^−2^) |  | High | 5 |
| *Bacteroidales* | −0.42  (2.66×10^−2^) |  | −0.37  (4.28×10^−2^) |  | −0.55  (1.67×10^−3^) | −0.49  (6.63×10^−3^) |  |  | High | 4 |
| *Prevotella* | −0.42  (2.64×10^−2^) |  |  | −0.51  (4.77×10^−3^) | −0.43  (1.80×10^−2^) |  |  |  | High | 3 |
| *Oscillospira* |  |  | −0.43  (1.91×10^−2^) |  | −0.38  (3.92×10^−2^) | −0.72  (1.01×10^−5^) |  |  | High | 3 |
| *Alphaproteobacteria* |  |  |  | −0.50  (5.47×10^−3^) |  | −0.57  (1.26×10^−3^) | −0.38  (4.45×10^−2^) |  | High | 3 |
| *Lachnospiraceae* |  |  |  | −0.39  (3.44×10^−2^) | −0.36  (4.88×10^−2^) | −0.47  (1.06×10^−2^) |  |  | High | 3 |
| SR1 |  |  | −0.41  (2.62×10^−2^) |  | −0.38  (3.92×10^−2^) |  |  |  | High | 2 |
| YS2 |  |  |  | −0.48  (7.84×10^−3^) |  | −0.44  (1.71×10^−2^) |  |  | High | 2 |
| *Ruminococcus* |  |  |  | −0.39  (3.74×10^−2^) |  | −0.55  (1.88×10^−3^) |  |  | High | 2 |
| *Prevotellaceae* |  |  |  |  |  |  | −0.49  (7.82×10^−3^) | −0.42  (2.59×10^−2^) | High | 2 |
| *Clostridiales* family XIII |  |  | −0.41  (2.44×10^−2^) |  |  |  |  |  | High | 1 |
| *Shuttleworthia* |  |  | −0.48  (6.83×10^−3^) |  |  |  |  |  | High | 1 |
| F16 |  |  |  | −0.46  (1.19×10^−2^) |  |  |  |  | High | 1 |
| *Prevotella ruminicola* |  |  |  |  | −0.40  (2.79×10^−2^) |  |  |  | High | 1 |
| *Veillonellaceae* |  |  |  |  | −0.47  (8.83×10^−3^) |  |  |  | High | 1 |
| *Oscillospira guilliermondii* |  |  |  |  |  | −0.37  (4.93×10^−2^) |  |  | High | 1 |
| *Blautia* |  |  |  |  |  | −0.65  (1.54×10^−4^) |  |  | High | 1 |
| *Eubacterium* |  |  |  |  |  |  | −0.48  (9.18×10^−3^) |  | High | 1 |
| *Quinella* |  |  |  | 0.49  (7.56×10^−3^) | 0.46  (1.04×10^−2^) |  | 0.44  (1.98×10^−2^) | 0.49  (7.66×10^−3^) | Low | 4 |
| *Prevotella bryantii* | 0.46  (1.35×10^−2^) |  |  |  |  | 0.56  (1.65×10^−3^) | 0.38  (4.36×10^−2^) |  | Low | 3 |
| *Olsenella* | 0.42  (2.62×10^−2^) |  |  |  |  | 0.58  (1.07×10^−3^) |  |  | Low | 2 |
| *Erysipelotrichaceae* | 0.41  (3.15×10^−2^) |  |  |  |  |  |  |  | Low | 1 |
| *Kandleria vitulina* | 0.60  (8.37×10^−4^) |  |  |  |  |  |  |  | Low | 1 |
| *Paludibacter* | −0.56  (2.12×10^−3^) | 0.38  (4.38×10^−2^) |  | −0.52  (4.10×10^−3^) |  |  |  |  | None | 3 |
| *Fibrobacter succinogenes* | 0.46  (1.44×10^−2^) | −0.46  (1.48×10^−2^) |  |  |  | 0.51  (4.77×10^−3^) |  |  | None | 3 |
| *Coprococcus* | −0.43  (2.17×10^−2^) |  |  |  | 0.42  (2.20×10^−2^) |  |  |  | None | 2 |
| RFN20 | −0.52  (4.27×10^−3^) |  |  |  |  |  |  | 0.49  (8.22×10^−3^) | None | 2 |
| Archaeal taxon | | | | | | | | | | |
| *Methanosphaera* | 0.55  (2.70×10^−3^) |  |  | 0.51  (5.61×10^−3^) |  | 0.43  (2.02×10^−2^) | 0.40  (3.35×10^−2^) |  | Low | 4 |
| *Mbb. gottschalkii* and relatives |  |  |  |  | 0.39  (4.32×10^−2^) |  |  |  | Low | 1 |
| Ciliate taxon | | | | | | | | | | |
| *Eudiplodinium* |  |  |  |  | −0.47  (8.81×10^−3^) | −0.40  (2.89×10^−2^) |  |  | High | 2 |
| *Isotricha* |  |  |  |  |  |  | −0.44  (1.58×10^−2^) | −0.37  (4.61×10^−2^) | High | 2 |
| *Entodinium* |  |  | −0.45  (1.15×10^−2^) |  |  |  |  |  | High | 1 |
| *Epidinium* |  |  | 0.46  (1.02×10^−2^) |  |  |  |  |  | Low | 1 |
| Anaerobic fungal taxon | | | | | | | | | | |
| *Piromyces* 3 |  |  | −0.38  (4.03×10^−2^) |  | −0.40  (2.81×10^−2^) |  |  |  | High | 2 |
| AL6 |  |  |  |  |  | 0.49  (8.13×10^−3^) | 0.58  (6.88×10^−4^) | 0.47  (1.07×10^−2^) | Low | 3 |
| *Neocallimastix* 1 |  | 0.44  (2.02×10^−2^) |  |  |  |  |  |  | Low | 1 |
