# Supplementary material for: Two Different Bacterial Community Types Are Linked with the Low-Methane Emission Trait in Sheep
Source: PLoS One. 2014 Jul 31;9(7):e103171. doi: 10.1371/journal.pone.0103171 (PMC4117531; doi:10.1371/journal.pone.0103171)
Supplement: Table S2 — Analysis of variance between samples grouping into the three different bacterial community types based on PAM clustering. Bonferroni-corrected p-values obtained by performing ANOVA for significance (at 0.05 criterion) of bacterial taxa between the community clusters defined by PAM and mean relative abundances of significant bacterial taxa in the samples grouping into clusters 1 [ruminotype H], 2 [ruminotype S], and 3 [ruminotype Q]. Only significant taxa that contributed in average ≥1% to the total bacterial community in at least one community type are reported. (DOCX) [file pone.0103171.s008.docx]

**Table S2.** **Analysis of variance between samples grouping into the three different bacterial community types based on PAM clustering.** Bonferroni-corrected *p*-values obtained by performing ANOVA for significance (at 0.05 criterion) of bacterial taxa between the community clusters defined by PAM and mean relative abundances of significant bacterial taxa in the samples grouping into clusters 1 [ruminotype H], 2 [ruminotype S], and 3 [ruminotype Q]. Only significant taxa that contributed in average ≥1% to the total bacterial community in at least one community type are reported.

| Taxon | *p*-value | average relative abundance [%] | | |
| --- | --- | --- | --- | --- |
|  |  | cluster 3 | cluster 1 | cluster 2 |
|  |  | [Q] | [H] | [S] |
| *Quinella* | 1.1 × 10^−82^ | 45.1 | 5.8 | 0.8 |
| Clostridiales | 8.1 × 10^−32^ | 3.6 | 7.3 | 4.0 |
| Ruminococcaceae | 1.9 × 10^−17^ | 3.3 | 7.1 | 4.7 |
| *Fibrobacter succinogenes* | 3.8 × 10^−17^ | 3.4 | 3.6 | 7.5 |
| Lachnospiraceae | 1.4 × 10^−15^ | 5.2 | 10.6 | 9.4 |
| Catabacteriaceae | 4.7 × 10^−14^ | 0.7 | 1.9 | 0.7 |
| *Prevotella* | 5.4 × 10^−12^ | 14.3 | 24.1 | 27.2 |
| *Sharpea azabuensis* | 4.8 × 10^−11^ | 1.2 | 1.2 | 6.5 |
| *Ruminococcus* | 2.2 × 10^−10^ | 0.8 | 1.6 | 1.0 |
| Bacteroidales | 5.0 × 10^−10^ | 7.3 | 12.5 | 13.9 |
| *Coprococcus* | 5.6 × 10^−9^ | 0.6 | 1.3 | 0.9 |
| YS2 | 6.1 × 10^−6^ | 0.5 | 1.1 | 0.4 |
| *Olsenella* | 1.0 × 10^−5^ | 0.1 | 0.1 | 1.0 |
| *Prevotella bryantii* | 4.3 × 10^−3^ | 0.7 | 0.4 | 2.8 |
| Alphaproteobacteria | 1.7 × 10^−2^ | 0.7 | 1.7 | 1.1 |
| *Kandleria vitulina* | 3.3 × 10^−2^ | 0.6 | 0.2 | 1.5 |
