# Supplementary material for: Two Different Bacterial Community Types Are Linked with the Low-Methane Emission Trait in Sheep
Source: PLoS One. 2014 Jul 31;9(7):e103171. doi: 10.1371/journal.pone.0103171 (PMC4117531; doi:10.1371/journal.pone.0103171)
Supplement: Text S1 — Detailed Materials and Methods. (DOCX) [file pone.0103171.s009.docx]

**Text S1**

**Detailed Materials and Methods**

*Measurement of CH_4_ yields from sheep*

The use of animals, including welfare, feeding, experimental procedures, and the collection of rumen samples used for this study, was approved by the AgResearch Grasslands Animal Ethics Committee (Application number 11975), and complied with the institutional Codes of Ethical Conduct for the Use of Animals in Research, Testing and Teaching, as prescribed in the New Zealand Animal Welfare Act of 1999 and its amendments. CH_4_ measurements were conducted with a total of 340 New Zealand sheep, born in 2009 and separated into four cohorts, over the year 2010 at the New Zealand Ruminant Methane Measurement Centre, AgResearch Ltd., Palmerston North, New Zealand. The facility comprises 24 respiration chambers for sheep, and the structure and operation of the facility has been described by Pinares-Patiño and Waghorn [1]. During the measurement periods, the animals were fed a pelleted lucerne (alfalfa) diet at 2.0 times of their maintenance requirements [2], delivered in equal size meals at 09:00 and 15:00 h. Intake was monitored by measured uneaten feed, and is expressed as dry matter intake (DMI) per day. Individuals in each cohort were randomly allocated to four measurement sets (each of 24 sheep to match the 24-chamber facility). Within each set, the animals were randomly allocated to the respiration chambers. Each cohort (C1 to C4) was measured in two independent measuring rounds (referred to as measuring rounds [a] and [b]): cohort 1 in March (C1a) and April (C1b), cohort 2 in May (C2a) and June (C2b), cohort 3 in July (C3a) and August (C3b), and cohort 4 in early (C4a) and late September (C4b). Randomization was carried out for each round of measurement and measurements were conducted following acclimatization to the feed and location for 3 weeks for round [a] and at least 2 weeks for round [b]. Measurements of feed intake were conducted with sheep housed in metabolism crates over three days (in a common holding area) and then over two days in the respiration chambers. Two consecutive days of CH_4_ emissions data were collected for each individual in each of the two measurement rounds, giving a total of four days of CH_4_ yield data (g CH_4_/kg DMI) from each animal [3]. Hereafter, the term CH_4_ yield refers to emissions in g CH_4_/kg DMI.

*Rumen sampling and sample processing*

At the end of each of the two CH_4_ measurement rounds, and immediately following the release of the sheep from the respiration chambers (17-18 h after the last feeding), a rumen sample (~50 g wet weight) was collected by stomach tubing and immediately stored at −20°C. In this way, a total of 680 rumen samples were collected; two per animal. Animals within each separate cohort were ranked on the basis of their 4-day average CH_4_ yield, and rumen samples from the highest (Hi) and lowest (Lo) emitters were selected for analysis of rumen microbial community structure (Table 1; File S1, column “AK(37), CH4Group”). In cohort 1, 15 of the 17 highest CH_4_ emitting animals and 13 out of the 15 lowest CH_4_ emitting animals were used. In cohort 2, the 15 highest and 15 of the 16 lowest CH_4_ emitting animals were used. Rumen samples from the 15 highest emitting animals and the 15 lowest emitting animals in cohorts 3 and 4 were used. This gave a total of 236 rumen samples (Table 2; for metadata of individual samples refer to File S1). All rumen samples were freeze-dried, homogenized using a 120 W household coffee grinder (CG2B, Breville), which was thoroughly wiped with 70% ethanol between samples, and then stored at −20°C.

*Volatile fatty acid analysis*

For volatile fatty acid (VFA) analysis, a 1.8-ml subsample of rumen contents was centrifuged at 20 000 *g* at 4°C for 5 minutes and 0.9 ml of the supernatant was added to 0.1 ml of acid solution containing 200 μl concentrated ortho-phosphoric acid per ml water and 20 mM 2-ethyl butyric acid. These amended samples were stored at −20°C [4]. For analysis, the stored samples were thawed, centrifuged again as described above, and the supernatant used to measure VFA concentrations by gas-liquid chromatography [4].

*Extraction of nucleic acids*

Nucleic acids were extracted from 30-mg of rumen sample with a combined bead-beating, phenol-chloroform and column purification protocol [5], using the QIAquick 96 PCR purification kit (Qiagen, Hilden, Germany). Cells were disrupted by bead-beating (FastPrep FP120; Qbiogene, Carlsbad, CA, USA; 45 s at 6.5 m s^–1^) with 550 μl phenol-chloroform-isoamylalcohol (25:24:1), 282 μl buffer A (200 mM NaCl, 200 mM Tris, 20mM EDTA, pH 8 with NaOH), 268 μl PM buffer (Qiagen), and 200 μl sodium dodecyl sulfate (20% wt/vol). After centrifugation at 20 000 *g* at 4°C for 20 min, the upper phase (350 μl) was removed, mixed with 650 μl PM buffer and processed through the QIAquick column by applying a vacuum with a QIAvac 96 vacuum manifold (Qiagen). All subsequent steps were carried out as recommended by the manufacturer, and DNA was eluted in 80 μl elution buffer (10 mM Tris, pH 8.5 with HCl).

*Enumeration of archaeal 16S rRNA genes using quantitative PCR*

The abundances of archaeal 16S rRNA genes were quantified in randomly-selected rumen samples of 12 Hi emitters and 12 Lo emitters of cohort 1, using a Rotor Gene 6000 real-time rotary analyzer (Qiagen) and amplicon detection by SYBR Green I fluorescence (LightCycler 480 SYBR Green I Master; Roche Diagnostics, Auckland, New Zealand). Reactions were set up in a Gene-Disc 100 (Qiagen) and sealed with permanent adhesive film (Qiagen). Archaeal 16S rRNA genes were amplified using the primers Ar915aF and Ar1386R as described previously [6]. Briefly, each reaction contained, in a total volume of 20 μl, 10 μl SYBR Green mix (Roche Diagnostics), 10 pmol of each primer, and 2 μl of template rumen or standard DNA. The balance was water. External standards were prepared by making 10-fold serial dilutions of purified plasmid DNA containing cloned marker loci amplified from DNA of rumen methanogens. The following temperature profile was used: 95°C for 10 min, followed by 50 cycles of 95°C for 10 s, 59°C for 5 s and extension at 72°C for 10 s. Melting curves between 72 and 98°C were evaluated after each run for each PCR to confirm the absence of unspecific signals. DNA amplification was performed on three different dilutions of the template, each in duplicate, to yield six replicates for each sample (1:10, 1:50 and 1:100), and only values that fell within the linear range of the standard curve were used in calculations (generally the 1:50 and 1:100 dilutions). Archaeal 16S rRNA gene copy numbers are reported as copies per gram of freeze-dried rumen contents.

*Amplification of bacterial, archaeal, ciliate and anaerobic fungal marker genes*

Primers used for PCR amplification of bacterial (primer pair Ba9F and Ba515Rmod1) and archaeal (Ar915aF and Ar1386R) 16S rRNA genes, ciliate 18S rRNA genes (RP841F and Reg1302R) and anaerobic fungal internal transcribed spacer 1 (ITS1; MN100F and MNGM2) contained the 454 Life Science (Branford, CT, USA) adapters A (5’-CCA TCT CAT CCC TGC GTG TCT CCG ACT CAG-3’) or B (5’-CCT ATC CCC TGT GTG CCT TGG CAG TCT CAG-3’) for Titanium sequencing, a 2-base linker sequence between the barcode and the group-specific primer [7], and a unique 12-base error-correcting Golay barcode attached to adapter A for sample identification (Integrated DNA Technologies Inc., Coralville, IA, USA; [8]). For each individual barcode, primer, and sample combination, a PCR master mix was prepared with 40 μl of *Taq* PCR MasterMix (Qiagen), 28 μl non-barcoded primer (to a final concentration of 0.2 μM) and 8 μl of barcoded primer (to a final concentration of 0.2 μM). An aliquot of 19 μl was transferred to serve as a no-template negative control. The remaining 57 μl were spiked with 120 ng of template DNA in 3 μl and then equally divided into three aliquots of 20 μl each, each of which was subjected to PCR amplification. Amplification was performed as follows on a thermocycler ProS (Eppendorf, Hamburg, Germany), with an initial denaturation at 95°C for 2 min, 30 cycles (or 35 cycles for anaerobic fungi) of denaturing (95°C, 20 s), annealing (52°C for Bacteria, or 59°C for Archaea, or 54°C for ciliate protozoa, or 50°C for anaerobic fungi; 20 s) and elongation (72°C, 1 min), and a final 7-min extension at 72°C. The triplicates of PCR amplicons were pooled, and the correct sizes of PCR products and the absence of amplicons in the negative controls were verified by agarose gel electrophoresis. To facilitate sample handling, the amplifications were performed in three batches of rumen DNA. Each batch was processed separately. For each batch, the amplicons of all four microbial groups were quantified using the Quant-iT dsDNA BR assay kit (Invitrogen, Carlsbad, CA, USA) and a fluorometer (BioTek Instruments, Winooski, VT, USA). Bacterial amplicons were pooled so that an equimolar amount of product from each sample was present in the final pooled sample. The same was done for the archaeal amplicons, ciliate protozoal amplicons and anaerobic fungal amplicons. A total of 1 μg DNA from each pool was loaded onto a 1%-agarose gel (wt/vol). Bands were visualized and excised under blue light transillumination, and amplicon pools were gel purified with the QIAquick gel extraction kit (Qiagen). Subsequently, gel-purified amplicon pools were quantified in triplicate with the Quant-iT dsDNA HS assay kit (Invitrogen). Emulsion PCR was performed with the Lib-L kit (Roche Diagnostics). DNA positive beads were enriched, counted on a Z1 particle counter (Beckman Coulter, Brea, CA, USA), and loaded onto a picotitre plate for pyrosequencing on a 454 Life Sciences Genome Sequencer FLX machine (Center for Genome Sciences and Systems Biology, Washington University of St. Louis, USA). The rumen DNA derived amplicons have the following naming convention: Microbial group abbreviation (“Ba.”=bacterial 16S rRNA, “Ar.”=archaeal 16S rRNA, “Pr.”=ciliate 18S rRNA, “Fu.”=anaerobic fungal ITS1), ruminant species abbreviation (“S”=sheep), AgResearch internal ruminant identifier code (up to 4 numbers), measuring round (e.g., “.C1aMar10”=Cohort 1, measuring round [a], March 2010), and a code for the emitter group (Lo or Hi).

*Phylogenetic assignment*

Sequence data were processed and analyzed following the procedure described by Caporaso *et al.* [9]. Sequence reads were assigned to corresponding samples by examining the 12-bp error-correcting Golay barcodes with default QIIME v1.5 parameters [9]. For bacteria and archaea, a highly stringent sequence quality assessment was performed by using only those sequences for further analyses that were ≥400 bp (including primers) in length and in which both the forward and reverse primers were detected (-z truncate_remove). Sequences stemming from ciliate 18S rRNA gene and anaerobic fungal ITS1 amplicons that were >200 bp in length were truncated to variable lengths so that the average quality score was >25 and only sequences without ambiguous characters were included in the analyses. An arbitrary cut-off of 97% was used to cluster sequence reads from bacteria and archaea into operational taxonomic units (OTUs), while the prefix/suffix OTU picking method was used for ciliate protozoa and anaerobic fungi [7]. For homopolymer-rich anaerobic fungal ITS1 sequences, the option –H 8 was passed to allow a maximum length of a homopolymer run of eight base pairs (default: –H 6). Sequence data were assigned to named taxa using BLAST against the greengenes database for bacterial 16S rRNA genes (gg_97_otus_4feb2011.fasta; [10]). Archaeal 16S rRNA genes [11], ciliate 18S rRNA genes [12], and anaerobic fungal ITS1 genes [13] were blasted against rumen specific, in-house databases derived from earlier studies (available from the authors upon request). Finally, the OTU abundance tables for all four microbial groups were summarized at the species- (bacteria), a mixed-taxon- (archaea, anaerobic fungi), or genus-level (ciliate protozoa). These sequence data have been submitted to the EMBL database under the study accession numbers ERP003779 (Bacteria), ERP003773 (Archaea), ERP003772 (ciliate protozoa), and ERP003764 (anaerobic fungi).

*Statistical analyses*

Significance of differences in CH_4_ yields between the Hi and Lo emitters were analyzed using the Student’s t-test with 2-tailed distribution and unequal variance in Excel (Microsoft Corp., Redmond, WA, USA).

Differences in archaeal 16S rRNA copy numbers in Hi and Lo emitters measured using quantitative PCR were determined by using the non-parametric Wilcoxon rank-sum test in R (www.r-project.org).

All analyses of high-throughput pyrosequencing data were carried out using the QIIME pipeline [9]. Resulting text files were imported into Excel and R for further statistical evaluation.

To estimate diversity in sheep rumen samples, Simpson’s indices of diversity (1-*λ*) were calculated individually for the bacterial, archaeal, ciliate and anaerobic fungal communities in each sample using the PAST software [14], based on Simpson’s index of dominance (λ). Simpson’s index of dominance (*λ*) was calculated according to the formula , where *n*_i_ is the number of individuals of species *i*, and *N* is the total number of individuals sampled [15]. Simpson’s index of diversity (1-*λ*) ranges from 0 to 1, with 1 indicating that no species are shared between the two communities and 0 indicating complete identity. First and third quartiles and medians for all measuring rounds were calculated, and box-and-whisker diagrams plotted in R, with the whiskers representing the minimum and maximum values excluding outliers.

For all analyses described below, taxonomic groups that did not contribute at least 1% to the community in at least one sample were excluded.

Correspondence analysis (CA) and principal coordinate analysis (PCoA) using Bray-Curtis dissimilarities were performed using the packages “ca” and “vegan” in R [16,17]. For bacteria, CA as well as PCoA showed gradual transitions between at least three bacterial community types. These types were distinguished in two different ways, 1. Samples were ranked based on CA coordinates (File S1, column “AL(38)”, CASerial”) and grouped manually (File S1, column “AM(39), CAType”), or 2. Samples were computationally divided into three clusters by Partitioning Around Medoids (PAM) based on Bray-Curtis dissimilarity (File S1, column “AO(41), PAMCluster”). Statistical differences between CH_4_ yields of the three community types were calculated based on both groupings and gave highly similar results.

Testing for statistically significant taxa between clusters identified using the two different grouping methods (by CA type and PAM cluster) was done by ANOVA using the otu_category_significance.py script in QIIME. Only taxa giving Bonferroni-corrected *p*-values ≤0.05 were regarded as being statistically significant between groupings.

Differences in microbial community structure between samples stemming from Lo and Hi CH_4_ emitting animals were also explored using canonical discriminant analysis and multivariate analysis of variance (MANOVA) using the “MASS” package in R for each microbial group individually [18]. Canonical discriminant analysis determines linear functions that provide maximum separation of groups of individuals while keeping the variation within groups as small as possible. For ciliate protozoa and anaerobic fungi, the plotted sample points from the CH_4_ groupings (Lo and Hi) overlapped, and so tests for normality or significance between CH_4_ phenotypes were not carried out.

*In-depth phylogenetic analysis of significant bacterial taxa*

The representative sequence of an OTU stemming from a bacterial group significantly correlated with either LM or HM was obtained and the taxonomic assignment obtained earlier by BLAST against the greengenes database [10] was manually added to the sequence identifier of the representative sequence. Sequences were aligned against the ARB SILVA v.108 database [19] using SINA aligner [20], imported into ARB [21] and calculated into a Neighbor-Joining reference tree using the ARB Fast Parsimony tool and *Escherichia coli* positions 62−514 to determine their phylogenetic position.

**References for Text S1**

1. Pinares-Patiño CS, Waghorn GC (2012) Technical manual on respiration chamber designs. http://www.nzagrc.org.nz/user/file/65/GRA-MAN-Facility-BestPract-2012-FINAL.PDF. DOA: 04 June 2014.

2. Freer M, Dove H, Nolan JV (2007) *Nutrient requirements of domesticated ruminants*. CSIRO Publishing, Collingwood, Victoria, Australia.

3. Pinares-Patiño CS, Hickey SM, Young EA, Dodds KG, MacLean S, et al. (2013) Heritability estimates of methane emissions from sheep. Animal 7: 316–321.

4. Sun XZ, Hoskin SO, Muetzel S, Molano G, Clark H (2011) Effects of forage chicory (*Cichorium intybus*) and perennial ryegrass (*Lolium perenne*) on methane emissions *in vitro* and from sheep. Animal Feed Science & Technology 166–167: 391–397.

5. Rius A, Kittelmann S, Macdonald KA, Waghorn GC, Janssen PH, et al. (2012) Nitrogen metabolism and rumen microbial enumeration in lactating cows with divergent residual feed intake fed high-digestibility pasture. Journal of Dairy Science 95: 5024–5034.

6. Jeyanathan J, Kirs M, Ronimus RS, Hoskin SO, Janssen PH (2011) Methanogen community structure in the rumens of farmed sheep, cattle and red deer fed different diets. FEMS Microbiology Ecology 76: 311–326.

7. Kittelmann S, Seedorf H, Walters WA, Clemente JC, Knight R, (2013) Simultaneous amplicon sequencing to explore co-occurrence patterns of bacterial, archaeal and eukaryotic microorganisms in rumen microbial communities. PLOS ONE 8: e47879.

8. Fierer N, Hamady M, Lauber CL, Knight R (2008) The influence of sex, handedness, and washing on the diversity of hand surface bacteria. Proceedings of the National Academy of Sciences USA 105: 17994–17999.

9. Caporaso JG, Kuczynski J, Stombaugh J, Bittinger K, Bushman FD, et al. (2010) QIIME allows analysis of high-throughput community sequencing data. Nature Methods 7: 335–336.

10. McDonald D, Price MN, Goodrich J, Nawrocki EP, DeSantis TZ, et al. (2011) An improved Greengenes taxonomy with explicit ranks for ecological and evolutionary analyses of bacteria and archaea. The ISME Journal 6: 610–618.

11. Janssen PH, Kirs M (2008) Structure of the archaeal community of the rumen. Applied & Environmental Microbiology 74: 3619–3625.

12. Kittelmann S, Janssen PH (2011) Characterization of rumen ciliate community composition in domestic sheep, deer, and cattle, feeding on varying diets, by means of PCR-DGGE and clone libraries. FEMS Microbiology Ecology 75: 468–481.

13. Koetschan C, Kittelmann S, Lu J, Al-Halbouni D, Jarvis GN, et al. (2014) Internal transcribed spacer 1 secondary structure analysis reveals a common core throughout the anaerobic fungi (Neocallimastigomycota). PLOS ONE 9: e91928.

14. Hammer O, Harper DAT, Ryan PD (2001) PAST: Palaeontological statistics software package for education and data analysis. Palaeontologia Electronica 4: 1–9.

15. Dollhopf S, Hashsham S, Tiedje J (2001) Interpreting 16S rDNA T-RFLP data: application of self-organizing maps and principal component analysis to describe community dynamics and convergence. Microbial Ecology 42: 495–505.

16. Nenadic O, Greenacre M (2007) Correspondence Analysis in R, with two- and three-dimensional graphics: The ca package. Journal of Statistical Software 20: 1–13.

17. Oksanen J, Blanchet G, Kindt R, Legendre P, Minchin PR, et al. (2008) Vegan: community ecology package, R package version 2.0-8. http://www.r-project.org.

18. Venables WN, Ripley BD (2002) *Modern Applied Statistics with S* (Springer).

19. Pruesse E, Quast C, Knittel K, Fuchs BM, Ludwig W, et al. (2007) SILVA: a comprehensive online resource for quality checked and aligned ribosomal RNA sequence data compatible with ARB. Nucleic Acids Research 35: 7188–7196.

20. Pruesse E, Peplies J, Glöckner FO (2012) SINA: Accurate high-throughput multiple sequence alignment of ribosomal RNA genes. Bioinformatics 28: 1823–1829.

21. Ludwig W, Strunk O, Westram R, Richter L, Meier H, et al. (2004) ARB: A software environment for sequence data. Nucleic Acids Research 32: 1363–1371.
