# Supplementary material for: Two Different Bacterial Community Types Are Linked with the Low-Methane Emission Trait in Sheep
Source: PLoS One. 2014 Jul 31;9(7):e103171. doi: 10.1371/journal.pone.0103171 (PMC4117531; doi:10.1371/journal.pone.0103171)
Supplement: Text S2 — Microbial community composition across all samples. (DOCX) [file pone.0103171.s010.docx]

**Text S2**

**Microbial community composition across all samples**

Microbial community diversity and composition in the 236 sheep rumen samples were analysed based on 16S rRNA (bacteria and archaea), 18S rRNA (ciliate protozoa), and internal transcribed spacer 1 (ITS1; anaerobic fungi) marker genes. Only full-length bacterial and archaeal sequencing reads were used for community analyses (average amplicon lengths without primer: 469 bp [Bacteria] and 450 bp [Archaea]).

After high quality-filtering, we used a minimum sequence read cut-off of 781 bacterial 16S rRNA gene sequence reads per sample (total reads: 396 196; mean: 1 723; range: 781 - 4 073) and obtained useable data from 230 of the rumen samples. Overall, nearly all the members of the bacterial communities belonged to only seven orders: Clostridiales (42.7%), Bacteroidales (38.8%), Erysipelotrichales (5.6%), Fibrobacterales (5.3%), Anaeroplasmatales (2.2%), an as yet unidentified order of Alphaproteobacteria (1.3%), and Coriobacteriales (1.0%; Figure S1A). A further 0.2% of sequencing reads were assigned to other known orders within the Bacteria, and 0.01% of sequences could not be assigned reliably.

Archaea were analysed using a minimum read number cut-off of 515 sequences per sample (total reads: 340 405; mean: 1 506; range: 515 - 3 545), resulting in data from 226 of the rumen samples. Archaeal communities were represented by only four major groups within the orders Methanobacteriales and Methanomassiliicoccales [1], previously also referred to as Methanoplasmatales [2], which together made up 99.9% of the total archaeal community across all of the rumen samples (Figure S1B).

Ciliate protozoa were sampled to a depth of at least 368 reads per sample (total reads: 323 463; mean: 1 376; range: 368 - 5 130), so that we had useable data from 235 of the rumen samples. The entodiniomorph genera *Epidinium* (49.1%) and *Entodinium* (34.5%) were the most dominant ciliate protozoa. Other known ciliate protozoa that were detected belonged to the genera *Eudiplodinium* (4.8%) and *Polyplastron* (3.9%) and to the holotrich genera *Dasytricha* (5.0%) and *Isotricha* (2.3%; Figure S1C).

For anaerobic fungi, the sequence read cut-off was set to 159 reads per sample (total reads: 123 814; mean: 534; range: 159 - 1 469), allowing us to include data from 232 of the rumen samples. Anaerobic fungal ITS1 sequence reads belonging to the groups JF423625 (52.5%), BlackRhino (18.6%), AL6 (16.3%), *Neocallimastix* 1 (5.5%), and *Piromyces* 3 (1.6%), and 4 (3.0%) represented the most common groups of anaerobic fungi in this study (Figure S1D). The as-yet undefined group JF423625 appears to be a distinct lineage that could be affiliated with the genus *Neocallimastix* (based on ITS1 primary sequence information alone; [3]) or to *Orpinomyces* (based on ITS1 primary sequence and secondary structure information; [4]).

**References for Text S2**

Iino T, Tamaki H, Tamazawa S, Ueno Y, Ohkuma M, et al. (2013) *Candidatus* Methanogranum caenicola: a novel methanogen from the anaerobic digested sludge, and proposal of *Methanomassiliicoccaceae* fam. nov. and *Methanomassiliicoccales* ord. nov., for a methanogenic lineage of the class *Thermoplasmata*. Microbes and Environments 28: 244–250.

Paul K, Nonoh JO, Mikulski L, Brune A (2012) "*Methanoplasmatales*", *Thermoplasmatales*-related archaea in termite guts and other environments, are the seventh order of methanogens. Applied and Environmental Microbiology 78: 8245–8253.

Kittelmann S, Naylor, GE, Koolaard JP, Janssen PH (2012) A proposed taxonomy of anaerobic fungi (Class *Neocallimastigomycetes*) suitable for large-scale sequence-based community structure analysis. PLOS ONE 7: e36866.

Koetschan C, Kittelmann S, Lu J, Al-Halbouni D, Jarvis GN, et al. (2014) Internal transcribed spacer 1 secondary structure analysis reveals a common core throughout the anaerobic fungi (Neocallimastigomycota). PLOS ONE 9: e91928.
