# Supplementary material for: Two Different Bacterial Community Types Are Linked with the Low-Methane Emission Trait in Sheep
Source: PLoS One. 2014 Jul 31;9(7):e103171. doi: 10.1371/journal.pone.0103171 (PMC4117531; doi:10.1371/journal.pone.0103171)
Supplement: Text S3 — Microbial community diversity across all samples. (DOCX) [file pone.0103171.s011.docx]

**Text S3**

**Microbial community diversity across all samples**

Simpson’s index of diversity (1-λ) was used to describe the diversity of microbial communities in all analysed rumen samples. This analysis suggested that the mean microbial community diversity varied strongly between individual samples, measuring rounds and cohorts for the four microbial groups analysed (Figure S2). Wilcoxon rank-sum tests were carried out between Simpson’s indices of diversity of the LM and the HM related samples in each measuring round. In two out of eight measuring rounds, a lower diversity within the bacterial communities was correlated with lower CH_4_ yields (data not shown). However, the samples from the remaining measuring rounds did not show any significant differences in community diversity between the LM and HM samples. We concluded that diversity did not explain differences between CH_4_ groupings. No significant differences in archaeal and fungal community diversity were detected between samples belonging to different CH_4_ groupings in any of the eight measuring rounds (data not shown). Within ciliate communities, a lower diversity was correlated with LM yields in only one out of eight measuring rounds (C1a; data not shown).
